# Data-centric annotation analysis for plant disease detection: Strategy, consistency, and performance

**DOI:** 10.3389/fpls.2022.1037655

**Published:** 2022-12-07

**Authors:** Jiuqing Dong, Jaehwan Lee, Alvaro Fuentes, Mingle Xu, Sook Yoon, Mun Haeng Lee, Dong Sun Park

**Affiliations:** ^1^ Department of Electronic Engineering, Jeonbuk National University, Jeonju, South Korea; ^2^ Core Research Institute of Intelligent Robots, Jeonbuk National University, Jeonju, South Korea; ^3^ Department of Computer Engineering, Mokpo National University, Muan, South Korea; ^4^ Fruit Vegetable Research Institute, Chungnam A.R.E.S, Buyeo, South Korea

**Keywords:** plant disease detection, annotation strategy, inconsistent bounding box, data-centric, noisy labels

## Abstract

Object detection models have become the current tool of choice for plant disease detection in precision agriculture. Most existing research improved the performance by ameliorating networks and optimizing the loss function. However, because of the vast influence of data annotation quality and the cost of annotation, the data-centric part of a project also needs more investigation. We should further consider the relationship between data annotation strategies, annotation quality, and the model’s performance. In this paper, a systematic strategy with four annotation strategies for plant disease detection is proposed: local, semi-global, global, and symptom-adaptive annotation. Labels with different annotation strategies will result in distinct models’ performance, and their contrasts are remarkable. An interpretability study of the annotation strategy is conducted by using class activation maps. In addition, we define five types of inconsistencies in the annotation process and investigate the severity of the impact of inconsistent labels on model’s performance. Finally, we discuss the problem of label inconsistency during data augmentation. Overall, this data-centric quantitative analysis helps us to understand the significance of annotation strategies, which provides practitioners a way to obtain higher performance and reduce annotation costs on plant disease detection. Our work encourages researchers to pay more attention to annotation consistency and the essential issues of annotation strategy. The code will be released at: https://github.com/JiuqingDong/PlantDiseaseDetection_Yolov5 .

## 1 Introduction

Plant disease recognition concerns many farmers and researchers in agriculture. Once a plant is affected by diseases, the damage can be easily propagated to the entire crop, causing several production and economic risks (Carroll et al., 2017). According to the statistics, plant diseases caused by bacteria, fungi, nematodes and viruses cost the global economy USD220 billion annually (Savary et al., 2019). Furthermore, plant disease negatively affects agricultural production, increasing the number of hungry people. Hunger-related fatalities reached 4 million in 2020, 10 times the number of COVID-19 fatalities in the same period, and most of them are distributed in less developed countries and regions (He and Krainer, 2020). If plant diseases are not discovered in time, food insecurity will increase. Therefore, plant disease identification has been a crucial issue in recent years.

Accurate and rapid plant disease detection algorithms can help reduce the risk of spreading plant pests and economic losses, which is also conducive to developing automatic agricultural production. Not only does the level of automation in agriculture require higher yields, but the quality is also a critical factor (Saleem et al., 2019; Vishnoi et al., 2021). To improve the quality of crops, protecting plants from potential diseases is crucial, which also helps reduce food production costs. Nevertheless, methods based on manual screening require high labor costs, and inspectors need to have relevant domain knowledge (Ferentinos, 2018). Therefore, designing an automated detection system for plant disease is meritorious and necessary.

**Figure 1:**
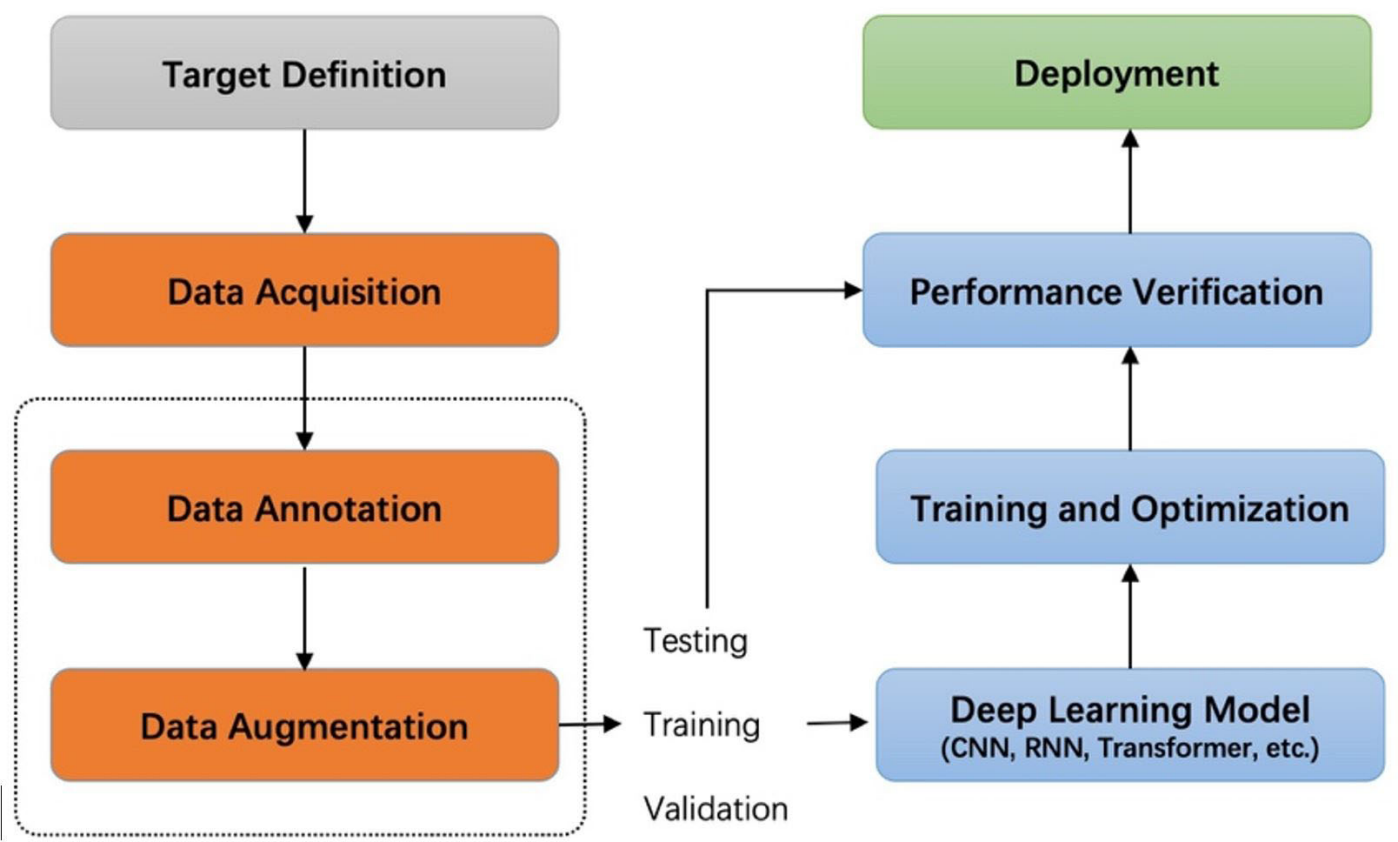
Pipeline of a deep learning project. The dashed box is the focus of our research.

Recently, image acquisition is becoming easier and easier in the agricultural field because of the increased use of cameras and sensors. Intelligent applications based on agricultural images have been widely used in many aspects of agriculture, such as plant disease and pest detection (Hughes and Salathé, 2015; Fuentes et al., 2017; Liu et al., 2017; Fuentes et al., 2018; Wiesner-Hanks et al., 2018; Parraga-Alava et al., 2019; Nazki et al., 2020; Li et al., 2020; Singh et al., 2020; Fuentes et al., 2021; Fenu and Malloci, 2021), fruit detection (Gao et al., 2020), yield prediction (Schauberger et al., 2020), visual navigation (Emmi et al., 2021), and agricultural robots (Chen et al., 2020). Deep learning technology has been playing a dominant role in various detection tasks. This technology can potentially reduce the negative impacts of plant diseases by promptly estimating the damage using non-intrusive sensors such as RGB cameras. Deep learning-based systems have achieved higher recognition and, at the same time, have contributed with environmental-friendly tools to perform plant state monitoring. Generally, supervised learning projects based on deep learning start by defining the target task, collecting and labeling data, training and optimizing the network, and finally deploying the model in practical scenarios for testing. **Figure 1** shows the pipeline of a general deep learning project. In this pipeline, data collection and labeling are upstream tasks, and training and optimization of models are downstream tasks. Our work studies the impact of the upstream task on downstream task performance.

Recent works on plant disease detection aim to design and optimize the network structure to improve the feature extraction capability. They assume that datasets are well-annotated and immutable, and ablation studies are implemented to indicate the efficiency of each module they proposed. In contrast to previous works, we argue that datasets with different annotation strategies will perform differently in a specific task. However, previous works (Hughes and Salathé, 2015; Fuentes et al., 2017; Liu et al., 2017; Fuentes et al., 2018; Wiesner-Hanks et al., 2018; Parraga-Alava et al., 2019; Nazki et al., 2020; Li et al., 2020; Singh et al., 2020; Fuentes et al., 2021; Fenu and Malloci, 2021) ignored the impact of the annotation strategy on the detection model. In addition, image classification systems recently made a giant leap with the advancement of deep neural networks, which require sufficient accurate labeled data to be adequately trained. Nevertheless, acquiring an accurately annotated dataset is not always feasible due to several factors. For example, practitioners without computer vision knowledge lack experience on how to annotate high-quality boxes, while annotators without domain knowledge are also difficult to annotate accurate object boxes. Due to these practical challenges, the actual labels often deviate from the ideal value, which leads to labels that are inconsistent with the instances. Even though extensive works of deep learning techniques under class noise exist (Li et al., 2020; Bernhard and Schubert, 2021; Mao et al., 2021; Xu et al., 2021), it mainly focuses on computer vision datasets such as MS-COCO (Lin et al., 2014), PASCAL VOC (Everingham et al., 2010), and ImageNet (Deng et al., 2009) rather than domain-specific datasets. In some domains, the definition of an object is significantly different from generic objects in these typical datasets, thus bringing annotation ambiguities. The literature lacks a specific study on inconsistent labels in plant disease detection.

Collecting images with labels is expensive and challenging in many scenarios. Many data augmentation algorithms have been proposed to alleviate this issue as effective and effcient policies (Cubuk et al., 2019). However, if the specificity of the task is not considered, implementing data augmentation may affect the performance gain brought by this technique. In the existing literature on data augmentation techniques, there is a lack of analysis of annotation inconsistency caused by the data augmentation process.

Consequently, in this work, data-centric machine learning (Miranda, 2021) is proposed to find efficient ways to construct suitable datasets to improve the performance of artificial intelligence models. Our main contributions are summarized as follows:

1. Four annotation strategies were proposed aiming to find ways to help practitioners to reduce labeling costs and improve plant disease detection performance through an efficient annotation strategy. To the best of our knowledge, there are no relevant studies on annotation strategies in plant disease detection.
2. We define five different types of noise to describe annotation inconsistency and investigate the severity of the impact on the model’s performance by perturbing clean bounding boxes. It is the first quantitative study of annotation consistency in plant disease detection.
3. We study annotation inconsistency caused by data augmentation in different scenarios. In addition, theoretical insights and empirical evaluations are provided to demonstrate the importance of annotation consistency to the model’s performance.
4. An interpretability study of the experimental results is conducted through the visualization method, which can help us analyze the logic behind recognizing novel data.

A detailed review of datasets and annotation, label consistency, and data augmentation for anomaly detection in plants and deep-learning techniques is presented in Section 2. Annotation strategies and quality analysis are detailed in Section 3. Section 4 shows the experimental results to demonstrate how annotation strategy and consistency affect the model’s performance. We found that using a symptom-adaptive strategy and adopting augmentation with a specific rotation angle results in better performance. Moreover, we use visualization techniques to display and analyze the features extracted by a convolutional neural network (CNN) under different labeling strategies. In the last section, we give some conclusions, which will guide subsequent annotation workers. Overall, this work lays a foundation for data annotation in intelligent agriculture, inspiring subsequent works in the related domains, and calling for the community to pay more attention to the essential issue of annotation strategies and quality.

## 2 Related work

In this section, we briefly review recent works related to the proposed approach. According to the constraints of the latest advances in plant disease recognition, there is no research work on annotation strategy. Accordingly, we analyze the logic behind constructing the dataset through examples presented in published papers. Regarding annotation consistency, we discuss noise-related deep learning methods for image classification and object detection. The related issues of data augmentation and deep-learning methods are discussed at the end of this section.

### 2.1 Datasets and annotation strategy

The construction of datasets is crucial for deep learning models. Data annotation is an integral part of dataset construction. Up to now, many datasets related to plant disease detection have been proposed. Plant Village dataset (Hughes and Salathé, 2015) was collected from field trials of crops infected with one disease. Firstly, the technicians collected leaves from the plant, then placed them against a surface with a grey or black background. From our observation, most images contain a whole leaf in a single frame. On the contrary, for crops such as corn and squash, the leaves were too large to capture in a single frame while retaining high-resolution, close-proximity views. In these cases, they took images of different sections of the same leaf. Li et al. (Li et al., 2020) used VGG16 and Inception-v3 models to identify different degrees of Ginkgo biloba diseases. Liu et al. (2017) proposed a new CNN structure to identify apple leaf disease. Parraga-Alava J et al. (Parraga-Alava et al., 2019) introduce a coffee leaf images dataset for classification and segmentation tasks. As with Plant Village, each image of the Ginkgo biloba dataset (Li et al., 2020), apple leaf dataset (Liu et al., 2017), and coffee leaf dataset (Parraga-Alava et al., 2019) contains only one leaf.

Singh D et al. (Singh et al., 2020) create the PlantDoc dataset for plant disease detection. While labeling the boxes, the authors made sure that the whole leaf should be present inside the box, and the size of the bounding box should not be smaller than approximately 1/8th of the image. Fenu G et al. (Fenu and Malloci, 2021) released a publicly available field-dataset collected to diagnose and monitor plant symptoms, called DiaMOS Plant. Wiesner-Hanks T et al. (Wiesner-Hanks et al., 2018) collected images of maize leaves taken in three ways: by a hand camera, with a camera mounted on a boom, and with a camera mounted on a small unmanned aircraft system. Each bounding box of DiaMOS Plant (Fenu and Malloci, 2021) and Corn2018 (Wiesner-Hanks et al., 2018) covers the entire leaf, but each image contains more than one box. Fuentes A (Fuentes et al., 2017; Fuentes et al., 2018; Fuentes et al., 2021). is one of the pioneers in plant disease detection. Unlike previous datasets, annotations in (Fuentes et al., 2018; Fuentes et al., 2021) are more complex because they allow one image or leaf to contain multiple classes of diseases.

Although there are many related works in plant disease detection, they ignore the discussion of annotation strategies. They focus on optimizing the model rather than the data preprocessing part. **Table 1** shows the details of the above datasets.

**Table 1:** Details of related datasets. Cls, Seg, and Det denote classification, segmentation, and detection respectively.

Datasets	Environment	Plant	Task	Images/Instances	Classes	Main annotation strategy
Plant Village(2015) (Hughes and Salathé, 2015)	Laboratory	Multiple	Cls	54309/-	38	One whole leaf per image
Ginkgo biloba(2020) (Li et al., 2020)	Laboratory and field	Ginkgo	Cls	3727/-	3	
Apple Leaf(2017) (Liu et al., 2017)	Laboratory	Apple	Cls	13689/-	4	
RoCoLe(2019) (Parraga-Alava et al., 2019)	Field	Coffee	ClsSeg	1560/-	4	
PlantDoc(2020) (Singh et al., 2020)	Internet	Multiple	Det	2598/9216	27	One whole leaf per bounding box, multiple bounding boxes per image
DiaMOS(2021) (Fenu and Malloci, 2021)	Field	Pear	Det	3505/-	6	
Corn2018(2018) (Wiesner-Hanks et al., 2018)	Field	Maize	Det	18222/105705	1	
Tomato(2018) (Fuentes et al., 2018; Fuentes et al., 2021)	Field	Tomato	Det	8927/49662	11	Part of a leaf or whole leaf per bounding box, multiple bounding boxes per image

### 2.2 Annotation consistency analysis

Real-world data is never perfect and often suffer from annotation consistency. Generally, annotation consistency will suffer from the degenerated label set, including wrongly assigned class labels and inaccurate, redundant, and missing bounding boxes, which can impact interpretations of the data, models created from the data, and decisions made based on the data. Hence, most current learning algorithms have integrated various approaches to improve their discriminative capability in noisy environments, but the degenerated label set can still introduce severe negative impacts.

Zhu X et al. (Zhu and Wu, 2004) differentiate noise into two categories: class noise and attribute noise. Then they analyze their impacts on the model’s performance separately. Flatow D et al. (Flatow and Penner, 2017) examine how sensitive the CNN model is to noise in the training set, particularly when the training set contains mislabeled or subjectively-labeled examples. They took an approach to simulate noise in the training set, which introduced a hyperparameter P to control the mislabeling proportion. Nazari Z et al. (Nazari et al., 2018) evaluated the class noise impact on the performance of three widely used machine learning algorithms namely discrete legendre transforms, support vector machine, and k-nearest neighbor. Xu M et al. (Xu et al., 2019) studied the missing instance-level label problem in object detection. They presented a novel framework that gives a trade-off between collecting fewer annotations and building a more accurate object detector. Liu et al. (2022) designed a novel noise-based data augmentation method to improve robustness for models towards unforeseen malicious inputs in black-box test settings.

Li Y et al. (Li and Chao, 2021) analyzed crop pest and disease datasets regarding data quality. The results showed that the selected good data with less quantity could reach the same performance with all training data in some recognition tasks. In other words, the limited good data can beat a lot of low-quality data. In addition, they found that high-quality data can bring about 10% to 20% performance improvement. Algan G et al. (Algan and Ulusoy, 2021) make a comprehensive survey of methodologies centered explicitly around deep learning in the presence of noisy labels. Most works focus on classification and object detection tasks on typical datasets rather than domain-specific ones.

In plant disease detection, the definition of the object is significantly different from generic objects in MS-COCO and PASCAL VOC, because the symptoms appear in a specific part of the leaf. In contrast, almost all bounding boxes in the MS-COCO dataset cover the complete target except for the incompletely displayed objects. However, there is no related work on noise impact for plant disease detection tasks.

### 2.3 Inconsistency from data augmentation

Data augmentation is usually used to solve shortages and unbalanced data problems. Almost all methods used data augmentation techniques to improve the feature diversity on plant disease recognition tasks, such as classification and detection. Xu M et al. (Xu et al., 2022) performed a comprehensive survey on image augmentation for deep learning with a novel informative taxonomy. They considered that algorithms should be split into three categories: model-free, model-based, and optimizing policy-based. The model-free category employs standard image processing methods such as geometry and color changes. Barbedo J G A et al. (Barbedo, 2019) explored using individual lesions and spots for the task rather than considering the entire leaf. Specifically, they expanded the original Plant Village dataset with various partitioning methods based on the symptoms of the disease. The extended dataset includes local disease areas such as a single lesion and clusters of lesions. However, there are some limitations to be aware of when using this method. For example, the cropped regional symptoms are more similar to other disease symptoms, which will mislead the model in judging the category. In contrast, a model-based method leverages trainable image generation models to achieve data augmentation. Xu et al. (2021) proposed a novel data augmentation paradigm that can adapt variations from one class to another. For example, a healthy tomato leaf image is translated into a powdery mildew leaf image. In this way, the data variation in the variation-minority classes is enlarged by the variation-majority class, which can improve the accuracy of abnormality recognition. However, such methods usually require two steps: the first step is generating the image, and the second is implementing the classification or detection task. The model’s performance for downstream tasks depends on the quality of the data augmentation of the generative model. Besides, the optimizing policy-based approach aims to find the optimal operations or their combinations. Some deep learning theories (Cubuk et al., 2019; Cubuk et al., 2020) were proposed to search for improved data augmentation policies automatically. However, regarding plant disease detection (Kobayashi et al., 2018; Afzaal et al., 2021), data augmentation strategies are empirical rather than based on an automatic search method. These works generally focus on methodological refinement but lack discussion of label consistency in the extended dataset. In real scenarios, original inconsistent labels are extended, and new inconsistent labels may also be generated when we implement data augmentation. This paper aims to study the impacts of inconsistent labels generated from data augmentation.

### 2.4 Data-centric deep-learning technics

CNN-based architectures in deep learning are proven to be the best in learning representations and solving complex computer vision and general artificial intelligence problems. Researchers revisited the architecture of CNNs throughout the recent years and proposed a series of famous works. A typical view is that the feature extractor’s capacity benefits from several factors, such as a wider and deeper network, a higher resolution input, and more powerful hardware (Tan and Le, 2019; Tan and Le, 2021). It is also applied to Transformer-based methods, which have been widely used in computer vision tasks (Liu et al., 2022). An interesting observation in (Tan and Le, 2019): with a similar architecture, by scaling the network depth, width, and input resolution, the image classification performance on ImageNet only improved by 2.4%, while the number of parameters increased from 22M to 208M. In (Liu et al., 2022), the cost becomes extensively huge, from 88 million increase to 3 billion. It is unwise to spend a considerable cost for a slight improvement in practical applications.

Data-centric deep learning approaches attempt to improve performance by analyzing the relationship between data quality, quantity, and networks. Some studies (Schmarje et al., 2022; Davani et al., 2022) have shown that inconsistent labels are unavoidable in the annotation process. Even where there is a single answer, disagreement among annotators is ubiquitous, making it difficult to decide on a gold standard. This disagreement may be generated from the annotator, the data, and the context (Basile et al., 2021). Reasonable use of noisy labels can make the model achieve the same performance as using clean labels (Uma et al., 2021). Therefore, the community also devotes itself to detecting and fixing inconsistencies generated by the data collection and annotation. Up to now, it remains an open issue. For example, a poor instance initialization could render failure during training (Liu et al., 2022). This conclusion is the same as our intuition: with the same data size, a clean dataset leads to better performance.

This paper is more concerned with the annotation strategy and consistency in object detection, which the above studies ignored. Data annotation strategies and consistency are crucial in object detection, affecting feature extraction performance from objects. To our best knowledge, there are no related works to analyze annotation strategy and consistency in plant disease detection.

## 3 Materials and methods

**Figure 2:**
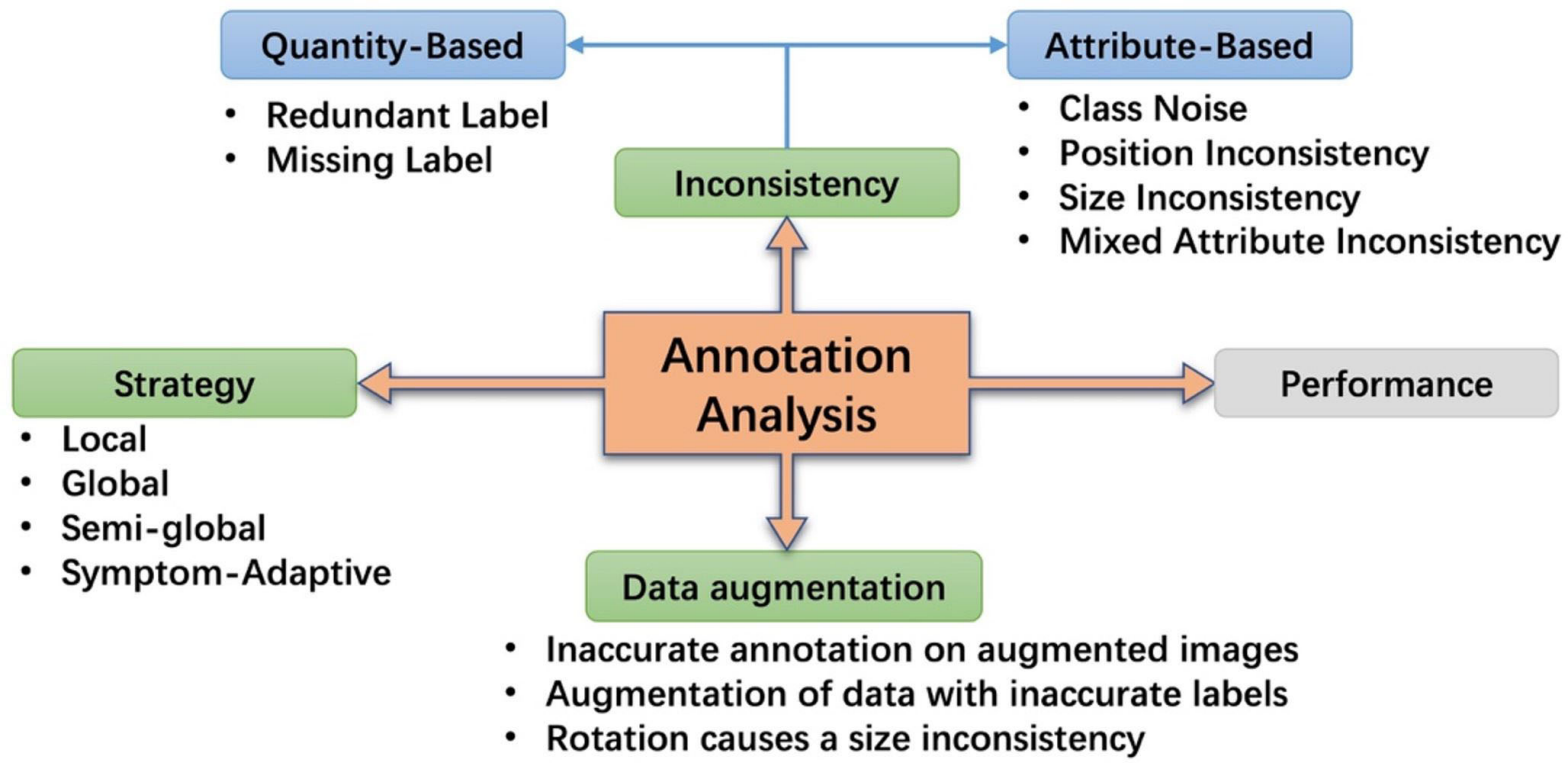
An overview of annotation analysis in this work. It contains four aspects: annotation strategy, annotation inconsistency, annotation inconsistency due to data augmentation, and performance.

Depending on the disease of the plant, the form of the disease appears in various ways in the plant. Some occur only in small parts of the leaf, and some occur in a wide range. In addition, some diseases can be locally observed and diagnosed, but some diseases can be diagnosed only when viewed in a wider area or even as a whole. Therefore, it is necessary to change the strategy of annotation according to the disease. At the same time, it should also be considered how these various annotation strategies will affect the performance of the detection system. In this section, we first introduce the datasets and propose four strategies to annotate plant disease regions. Furthermore, we redefine the concept of annotation inconsistency related to plant disease detection tasks. To explore the robustness of the neural network to inconsistency, noisy bounding boxes are simulated by perturbing the clean ones. As a complement, we study the annotation inconsistency due to data augmentation in real scenarios. An overview of the annotation analysis involved in this work is shown in **Figure 2**.

### 3.1 Dataset

**Figure 3:**
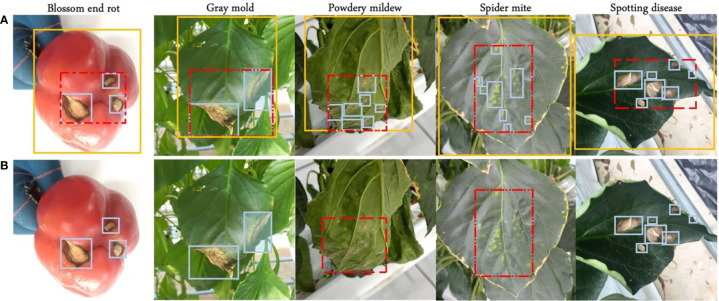
Four annotation strategies. **(A)** Blue boxes, red dashed boxes, and yellow boxes represent local, semi-global, and global labels, respectively. **(B)** Symptom-adaptive annotations are shown. Diseases with specific symptoms are labeled by semi-global boxes, while others are labeled by local boxes.

In this paper, paprika is used as a target plant for annotation analysis in plant disease detection, and its target diseases are four leaf diseases, gray mold, powdery mildew, spider mite, and spotting disease, and one fruit disease, blossom end rot. Plants are cultivated in controlled greenhouse environments. Paprika is used because is a target crop in our project. The dataset consists of 5928 paprika images. The samples of leaf diseases were collected in the well-lit greenhouse during the morning time, while fruit disease was collected in the well-lit greenhouse and laboratory. The dataset was collected in batches over the entire plant growth cycle by different mobile devices and other digital cameras to ensure data diversity. Resolutions range from 320x320 to 3024x4032. In addition, our dataset is much more complex because more than one disease may appear on a single leaf. Experts provide plant disease domain knowledge, and images are annotated by annotators and finally corrected by experts. After the annotation, the domain experts observed that some blurry instances should not have been annotated because they cannot fully guarantee the accuracy of these instances. In other words, these instances have a low confidence score. Experts also ensure the accuracy of the bounding box through careful screening and inspection. Finally, the blurry instances were filtered out to analyze the impact on model performance. Samples and annotation strategies are shown in **Figure 3**. More details are shown in **Table 2**, where the number of different disease instances is imbalanced.

**Table 2:** Details of bounding box under different annotation strategies.

Category	Images	Number of Bounding Boxes			
		Local	Semi-global	Global	Symptom-adaptive
Blossom end rot	1183	1577 + 39	1243 + 32	1243 + 32	1577 + 39
Gray mold	441	662 + 47	598 + 28	598 + 28	662 + 47
Powdery mildew	416	1372 + 96	417 + 25	417 + 25	417 + 25
Spider mite	420	2180 + 245	1239 + 93	1239 + 93	1239 + 93
Spotting disease	3468	9525 + 347	4021 + 122	4021 + 122	9525 + 347
Total	5928	15316 + 774	7518 + 300	7518 + 300	13420 + 551

C+R is used to denote the total number of labels, where C contains clear instances and R contains blurred instances used as redundant labels in Section 3.3.1.

### 3.2 Annotation strategy

In this work, the performance of neural networks to detect plant diseases under different annotation strategies is explored. A publicly available toolbox called ‘Labelimg’ is used to annotate our dataset. We consider four annotation strategies for all categories depending on the region and symptom: local label, global label, semi-global Label, and symptom-adaptive label, as shown in **Figure 3**.

#### 3.2.1 Local label

Intuitively, humans expect the bounding box to cover the object as tightly as possible. In this case, we annotate suspicious areas by using a small bounding box, and the background area in a box is much less. This method leads the neural network to focus more on learning the feature representation of the disease area, which can increase the inter-class feature distance. For instance, in powdery mildew and spider mite diseases, the boundary of pathological features is hard to distinguish for labelers, which will significantly challenge keeping annotation consistency. Thus, we assume that too many bounding boxes not only increase the annotation cost, but may also cause problems in annotation consistency, affecting the model’s performance.

#### 3.2.2 Global label

In this case, bounding boxes cover the whole fruit or leaf and include more background and suspicious instances. This is hard to focus on extracting the feature of the disease region for CNNs. Furthermore, when multiple diseases are on a leaf, it cannot simultaneously assign multiple labels to the same leaf. As shown in **Table 1**, in the dataset based on the global labeling strategy, each leaf contains only one disease. When two categories of global labels overlap, the detection capacity of CNNs may be weakened. This overlap may interfere with the feature extractor for classification, reducing the inter-class distance. Nevertheless, the global label annotation strategy is more widespread because it does not need to distinguish disease boundaries. When a leaf contains only one disease class and symptoms vary significantly from disease to disease, the global label is sufficient for the model to learn representative features. However, real-world scenarios are always unpredictable. For instance, a leaf may suffer from more than one disease. Therefore, global labels cannot meet the needs of complex scenarios.

#### 3.2.3 Semi-global label

We operate a larger bounding box than local labels to annotate the suspicious area. A semi-global bounding box merges local labels of the same class in one leaf, which leads to a bounding box that may include more than one instance of the same class. As a result, a bounding box contains the disease area and more background. Although more background in one box may affect the model’s performance in extracting disease features, the semi-global label strategy significantly reduces the annotation cost and improves annotation consistency. It does not need to distinguish the boundaries of multiple suspicious regions. Annotation under the semi-global is beneficial to extract more representative features and improve the performance for specific diseases due to better annotation consistency, even if it includes some background information.

#### 3.2.4 Symptom-adaptive label

Although local labels are more conducive to the neural network to learn the characteristics of lesions, annotation with a single strategy is problematic in meeting the demand for labeling complex datasets. As mentioned above, even an experienced annotator cannot distinguish the boundary of certain diseases, resulting in many noisy labels. Barbedo J G A et al. (Barbedo, 2019) divided symptoms into five categories: scattered small, scattered large, isolated, widespread, and powdery. They use different tailoring methods for diseases with various symptoms to implement data augmentation, which improves the accuracy of disease classification. Inspired by their work, we believe that the annotation strategies employed by different symptoms also should be different. In this work, a principle is proposed to divide symptoms into two categories: boundary-separable and boundary-inseparable.

● For a leaf with a clear color difference between the abnormal and normal area, and with a distinguishable boundary, we consider it belongs to boundary-separable diseases.
● Suppose the color of the lesion area gradually fades from center to edge of the leaf, and the color is similar to the normal area. In this case, we consider it belongs to boundary-inseparable diseases.

Specifically, in the paprika dataset, blossom end rot, gray mold, and spotting disease belong to boundary-separable diseases because their lesions are distinct from healthy areas. On the contrary, in powdery mildew and spider mite, it is hard to demarcate the boundaries of instances of those classes to annotate precisely. Therefore, these two categories belong to boundary-inseparable diseases. A local label strategy is performed for boundary-separable diseases, which helps the neural network better learn the lesion area’s features. Simultaneously, a semi-global label strategy is performed for boundary-inseparable diseases, which avoids potential problems caused by labeling inconsistencies. It is worth noting that, like the local labeling strategy, the semi-global labeling strategy also allows two or more diseases to appear on a leaf.

### 3.3 Annotation inconsistency

**Figure 4:**
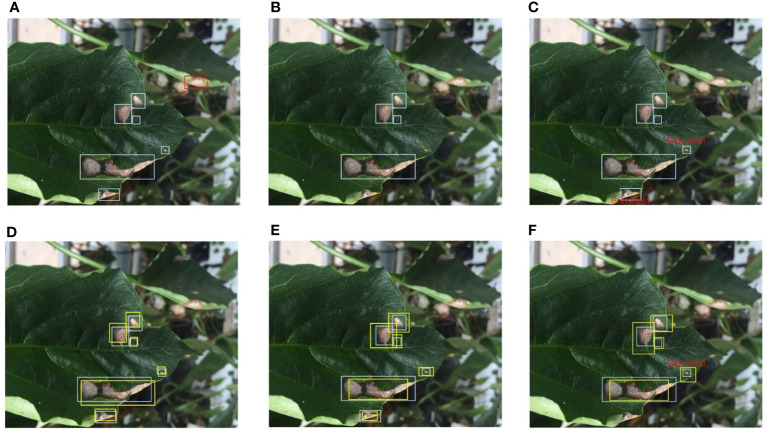
Different types of annotation inconsistencies. Blue bounding boxes represent the ground truth. **(A)** Redundant labels. The red dashed box includes a blurry instance. **(B)** Missing labels. Three bounding boxes are missing. **(C)** Class noise. There are two instances assigned the wrong class label. **(D)** Position inconsistency. The yellow bounding boxes deviate from the correct position. **(E)** Size inconsistency. The yellow bounding boxes do not cover the actual object as tight as possible. **(F)** Mixed attribute inconsistency. A mix of three inconsistencies, class noise, position inconsistency, and size inconsistency. Best view in color.

Fully supervised object detection (FSOD) requires that each instance should be annotated by an accurate bounding box. However, affected by empirical and systematic errors, the quantity and attributes of the factual bounding boxes in the label set cannot accurately represent all instances. In studies (Zhu and Wu, 2004; Flatow and Penner, 2017; Nazari et al., 2018), this issue is called label noise, while in this paper, call it annotation inconsistency. From a novel perspective, class noise is one of the annotation inconsistencies. Although CNNs are relatively robust to class noise, it is worthwhile to investigate in a more comprehensive study of inconsistency. We define five different types of inconsistency existing in real scenarios according to the practical task of plant disease detection into quantity-based inconsistency and attribute-based inconsistency. **Figure 4** shows examples of several different types of inconsistency.

#### 3.3.1 Quantity-based inconsistency

Due to subjective or objective factors, the number of bounding boxes deviates from the correct during the annotation process. We call quantity-based inconsistency. It mainly includes redundant labels and missing labels.

**Redundant Label.** A redundant label is used to describe an instance that should not have been labeled or is labeled multiple times. In a plant disease detection task, some blurred disease areas are generated due to the focus problem of the camera. These blurry disease areas are sometimes annotated. In this paper, we refer to a bounding box that includes a blurry instance as a redundant label. An example of a redundant label is shown in **Figure 4A**.

**Missing Label.** Satisfactory results have been achieved on large-scale detection benchmarks by FSOD methods. FSOD assumes that every example belonging to the target class should be annotated. A missing label is used to describe an instance that is ignored. The missing label profoundly affects the performance of FSOD methods. We also focus on studying the relationship between missing labels and the model’s performance on plant disease detection. An example of a missing label is shown in **Figure 4B**.

#### 3.3.2 Attribute-based inconsistency

Bounding boxes have several representations in object detection tasks. The VOC-based representation use (*c*,*x*_*min*_,*y*_*min*_,*x*_*max*_,*y*_*max*_) o represent the box’s properties. In the COCO-based representation method,.s used to represent the box’s attribute, where.epresent the category, coordinates of the upper left corner, and coordinates of the lower right corner of the box, respectively. In YOLO-based methods, *(c, x, y, w, h)* enotes the attribute of the bounding box, where.enotes the coordinates of the center point, and *(w, h)* epresents the width and height of the bounding box. All these methods explicitly or implicitly represent the box category, position, and size. Due to random factors and human error, instances are assigned a bounding box with inaccurate attributes. In this paper, three kinds of attribute inconsistencies are defined: class noise, position inconsistency, and size inconsistency.

**Class noise.** A universal noise is assigning an instance with a wrong category, which called class noise. This paper only discusses of the impact of random class noise on the model’s performance rather than empirical errors. An example of class noise is shown in **Figure 4C**.

**Position inconsistency.** Human annotators are accustomed to using the center point to represent the position of an instance. Also, the bounding box’s centric coordinates are crucial for the model to regress the target’s position. In this paper, position inconsistency refers to the degree of centric coordinate deviation between noisy ground truth and original ground truth. An example of position inconsistency is shown in **Figure 4D**.

**Size inconsistency.** The size of a bounding box determines how well the frame fits the target. Just as a person needs to wear well-fitting clothing, each disease area needs to be allocated a reasonably sized bounding box. In this paper, size inconsistency is used to describe that a bounding box does not match the size of the instance. An example of size inconsistency is shown in **Figure 4E**.

### 3.4 Inconsistency from data augmentation

Data augmentation is widely used in machine learning as an essential technique to improve performance. It usually consists of two parts: image augmentation and label augmentation. Sort by order, data augmentation can be divided into two cases:

**Case 1.** First, images are augmented and divided by version. Then, labelers annotate various versions of the image. In this case, data augmentation may lead to contradictory examples, which cause the same instance may be labeled with a different category, position, and size. Meanwhile, it will increase a considerable annotation cost.

**Case 2.** In the first step image annotation is conducted, and then followed by data augmentation. This way is preferred in most cases because it can save annotation cost. Nevertheless, in this case, inconsistent labels are also extended while implementing the data augmentation, which can lead to persistent misjudgment of some examples by the network.

**Figure 5:**
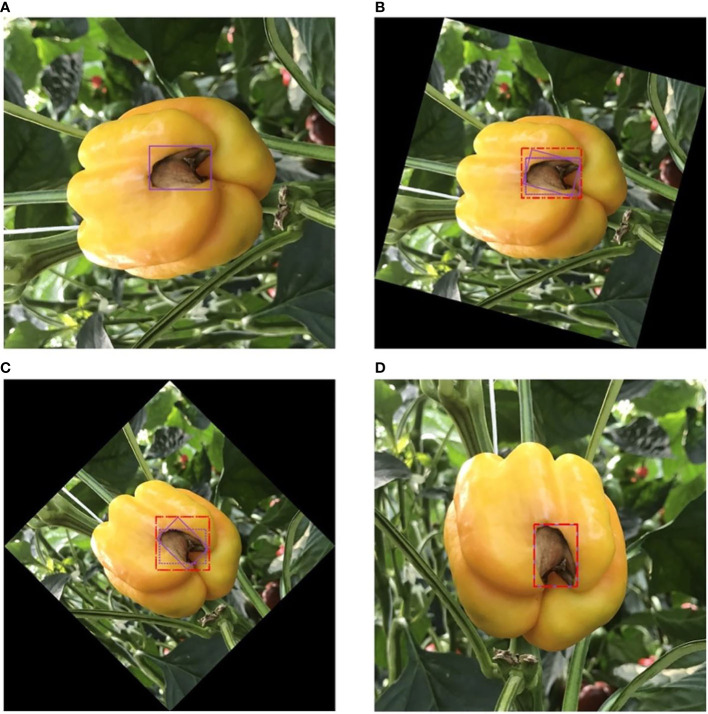
Size inconsistency from rotation. The purple solid line box represents the label box of the original image; the red dotted line box represents the real position of the label box after rotation; and the purple dotted line box represents the correct position of the label box after rotation. In the range of 0°-45°, the larger the rotation angle, the larger the size deviation. **(A)** 0°. **(B)** 15°. **(C)** 45°. **(D)** 90°.

We investigate the impact of inconsistency on the performance gain due to data augmentation. In addition, we observed that some geometric-based data augmentation methods change the size consistency of the labels. For example, rotation and cropping are the most common geometry-based data augmentation methods. The bounding box is automatically adjusted to fit the instance correctly when the image is cropped. In contrast, random rotation can cause the size of the bounding box to deviate from the correct and cannot be fixed automatically. As a complement, we investigate the impact of inconsistency on the performance gain due to random rotation. **Figure 5** shows the size inconsistency caused by rotation.

### 3.5 Evaluation metrics

The performance of the bounding box detector is evaluated using the following metrics:

**Intersection-over-Union metric (IoU):** We utilized a threshold of 0.5 to capture true positive detections generated by the model, as:


(1) $$IoU=\left|\frac{A\cap B}{A\cup B}\right|$$

where A and B represent the ground-truth and predicted box, respectively.

**Mean Average Precision score (mAP):** mAP is the area under the precision-recall curve calculated for all classes.


(2) $$AP=\frac{1}{11}\sum_{r\in \left[0,0.1,\ldots ,0.9,1\right]}P\left(r\right)$$

(3) $$P\left(r\right)=\underset{\tilde{r}:\tilde{r}\geq r}{\max}p\left(\tilde{r}\right)$$

where, *P(r)* s the maximum precision for any recall values greater than r, and $p\left(\tilde{r}\right)$ s the measured precision at recall $\tilde{r}.$

## 4 Experiments and results

### 4.1 Implementation

**Figure 6:**
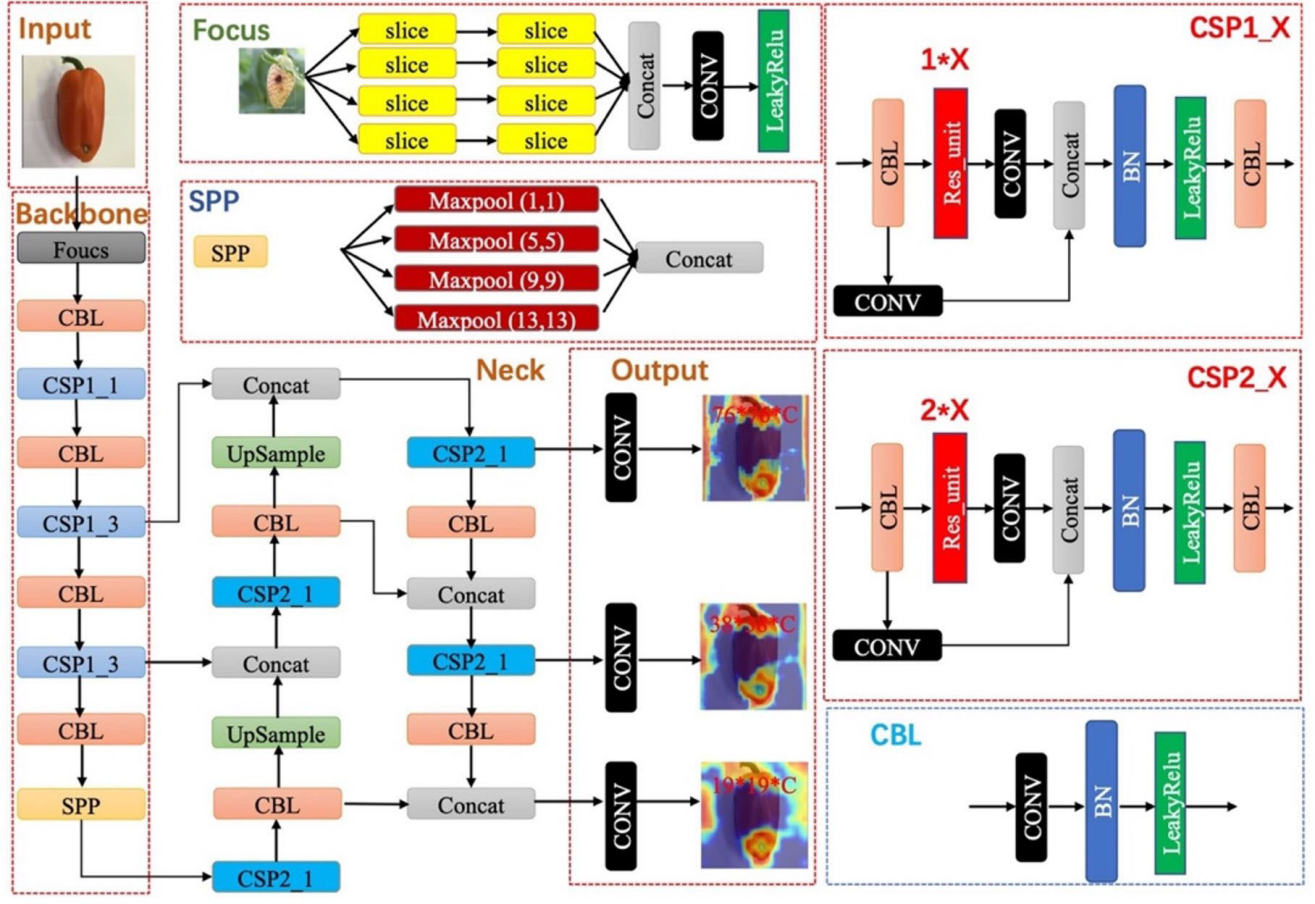
The architecture of YOLO-v5. The individual modules included are explained in the diagram.

Experiments are performed on our paprika disease dataset, which includes five annotated disease categories. As mentioned in the previous section, images in our dataset are fixed, while trainable instances are different among annotation strategies. Our dataset was divided into 80% training set, 10% validation set, and 10% testing set in all experiments. Training is proceeded on the training set, and then the evaluation is performed on the validation set. When the experiments achieve the expected results, the fnal evaluation is done on the testing set. In experiments related to data augmentation, data augmentation is only performed on the training set and validation set, ensuring that the test set is fixed. YOLO-v5 is a representative work of real-time detection in the industry. Therefore, we evaluate the effectiveness of different annotation strategies based on the YOLO-v5 model. The architecture and the main modules of YOLO-v5 are shown in **Figure 6**. To evaluate our methods, YOLO-v5 was trained and tested on 3 GTX 3090 GPUs and implemented in PyTorch 1.10.1.

### 4.2 Annotation strategy

The paprika disease dataset is used to evaluate four annotation strategies proposed for all categories. The performances of the different annotation strategies are shown in **Table 3A**. We train the dataset using YOLO-v5 architecture with different scales, from small to extra-large. A smaller model means that the neural network contains fewer layers and channels. As a result, a smaller model runs faster and requires less computation than a larger model. On the contrary, a larger model has more robust feature representation capabilities but requires more computation. In addition, for a rigorous conclusion, we employ a ten-fold cross-validation method for evaluation. However, we only validate four different annotation strategies on the YOLO-v5-Extra-large model due to the huge computational cost of cross-validation. Following the evaluation method in (Rafało, 2022), we provide the mean precision, standard deviation, minimum, and maximum for each disease. Additionally, we compute the median of the mAP. **Table 3B** shows the detailed statistical test results of ten-fold cross-validation.

**Table 3:** (A) Result of four different annotation strategies trained on various scales of YOLO-v5. L, SG, G, and SA denote local, semi-global, global, and symptom-adaptive respectively.

(A)	YOLO-v5-Small				YOLO-v5-Middle				YOLO-v5-Large				YOLO-v5-Extra-large			
	L	SG	G	SA	L	SG	G	SA	L	SG	G	SA	L	SG	G	SA
Blossom end rot (%)	91.9	87.2	83.6	**92.1**	91.7	90.5	84.3	**93.7**	92.6	90.9	88.4	**93.9**	94.2	91.6	86.9	**94.3**
Gray mold (%)	88.5	80.3	79.3	**89.3**	87.7	82.1	78.6	**90.8**	89.6	82.6	80.6	**90.6**	89.0	84.4	83.1	**90.8**
Powdery mildew (%)	62.1	82.8	80.2	**83.1**	66.3	83.6	79.8	**84.7**	63.9	84.5	79.7	**87.1**	65.3	**85.7**	**85.7**	85.3
Spider mite (%)	62.5	78.9	76.9	**82.0**	68.6	**84.9**	77.1	84.5	67.0	**86.6**	75.6	85.6	65.9	84.8	78.1	**88.6**
Spotting disease (%)	91.0	79.8	79.6	**93.0**	93.2	83.9	81.6	**93.8**	93.9	84.0	80.2	**94.3**	**94.6**	84.5	78.6	94.0
mAP@0.5 (%)	79.2	82.0	79.9	**87.9**	81.5	85.0	80.3	**89.5**	81.4	85.7	80.9	**90.3**	81.8	86.2	82.5	**90.6**
Parameters (M)	7.2				21.2				46.5				86.7			
FLOPs (B)	16.5				49.0				109.1				205.7			
(B)	YOLO-v5-Extra-large *															
	Local				Semi-global				Global				Symptom-adaptive			
Blossom end rot (%)	**93.8 ± 0.6 (92.9~94.7)**				91.1 ± 0.9 (89.9~93.1)				86.4 ± 0.7 (85.2~87.6)				93.7 ± 0.4 (93.4~94.6)			
Gray mold (%)	89.2 ± 1.2 (87.6~91.2)				84.1 ± 1.2 (82.5~86.4)				83.6 ± 1.1 (81.0~85.7)				**92.1 ± 0.9 (88.9~92.1)**			
Powdery mildew (%)	68.1 ± 1.5 (65.1~71.6)				85.6 ± 1.0 (83.4~87.3)				85.5 ± 0.8 (84.0~87.2)				**85.7 ± 0.9 (83.8~87.1)**			
Spider mite (%)	66.0 ± 1.9 (62.9~69.7)				86.0 ± 0.9 (85.1~88.2)				78.9 ± 2.0 (75.9~83.4)				**88.5 ± 1.2 (86.1~90.4)**			
Spotting disease (%)	**94.3 ± 0.6 (93.4~95.1)**				84.9 ± 1.1 (82.9~86.4)				78.8 ± 1.4 (76.4~82.3)				94.1 ± 0.4 (93.4~94.7)			
mAP@0.5 (%)	82.2 ± 0.7 (81.2~83.4)				86.4 ± 0.5 (85.4~87.2)				82.6 ± 0.7 (81.7~84.1)				**90.4 ± 0.4 (89.4~91.3)**			
Median of mAP@0.5 (%)	82.2				86.3				82.5				**90.4**			

* The values inside of the parenthesis represent the minimum and maximum values. Ten-fold cross-validation result of four different annotation strategies trained on YOLO-v5-Extra-large model. Bold fonts and background indicate the best performance (Maximum).

The result indicates that the performances of the same model are different by using distinct annotation strategies. From the view of diseases, as for blossom end rot, gray mold, and spotting disease, compared to larger bounding boxes containing more background, we found that the best results were obtained when adopting the local label strategy. The residual theory argues that the fewer healthy regions (background) a bounding box contain, the greater the inter-class distance of features, which also indirectly supports our results. However, powdery mildew and spider mite do not follow this theory. We consider that this phenomenon is symptom-related. Specifically, the diseased area of the former contrasts significantly with the green background, which is much easier to distinguish for CNNs. On the contrary, it is hard to distinguish the boundary of suspicious areas for powdery mildew and spider mite because of small regions and discontinuous distribution. Therefore, it significantly challenges the labeler to keep annotation consistency.

The result also shows that the mAP@0.5 of powdery mildew and spider mite significantly improved by adopting the semi-global bounding box for annotation. An important reason is that a semi-global label strategy avoids this tricky problem by merging suspicious regions. It makes the trade-off better consistency and less background. Also, it helps decision-makers reduce labeling costs. Moreover, we found that labeling methods using a single strategy can perform well in specific classes, but fail to generalize to all classes. Symptom-adaptive annotation strategies define a specific strategy for each disease based on symptoms, which ensures that each bounding box can be utilized efficiently.

From a perspective of model size, we usually get the best performance at the extra-large YOLO-v5 model. From small to extra-large model, the performance is only improved by around 3%, while the floating-point operations per second (FLOPs) and parameters considerably increase by approximately twelve times. Especially from middle to extra-large size, the model’s performance is only improved by around 1%. However, adjusting the annotation strategy improves the model’s performance by more than 9%. According to this observation, we believe that an effective annotation strategy can reduce the cost of annotation and significantly improve the model’s performance. Therefore, it is worth exploring how to design a more efficient annotation scheme for agricultural plant disease detection.

### 4.3 Annotation inconsistency

In this part, we analyze the impact of inconsistency on the model’s performance, including redundant labels, missing labels, class noise, position inconsistency, and size inconsistency. We show the results of the YOLO-v5-x model under the symptom-adaptive label strategy. We treat the annotated boxes of blurred instances as redundant labels. The number of blurred instances for each disease is shown in **Table 2**. **Table 4** shows the comparison of model’s performance on the label set with and without the redundant label. As for missing labels and class noise, we randomly perturb or remove the bounding box, where the probability ranges from 5% to 50%. In YOLO-based methods, *(c, x, y, w, h)* enote attribute of a bounding box, where *(x, y)* enotes the coordinates of the center point, and *(w, h)* epresents the relative width and height of the bounding box. ***I*** used to indicate the deviation degree of position inconsistency and size inconsistency, where**. *I*** range from 5% to 50%. The perturbing method is shown in Eq. 4. In this way, we can analyze whether the different types of inconsistency have the same impact on each disease. Finally, we add three attribute noises simultaneously, which we call mixed attribute inconsistency.


(4) $$\{\begin{array}{c} x_{inc}=x\pm 0.5*∆x*w,\;y_{inc}=y\pm 0.5*∆y*h\\ w_{inc}=w\pm ∆w*w,\;h_{inc}=h\pm ∆h*h \end{array}$$

**Table 4:** Comparison of with(w) or without(w/o) redundant label.

Category	w Redundant Noise	w/o Redundant Noise
Blossom end rot (%)	91.2	**94.3** (+3.1)
Gray mold (%)	84.8	**90.8** (+6.0)
Powdery mildew (%)	80.9	**85.3** (+4.4)
Spider mite (%)	82.1	**88.6** (+5.5)
Spotting disease (%)	91.5	**94.0** (+2.5)
mAP@0.5 (%)	86.1	**90.6** (+4.5)

Bold fonts indicate the best performance in comparative experiments (Maximum).

where *x*_*inc*_,*y*_*inc*_,*w*_*inc*_,*h*_*inc*_ enote the attribute of a bounding box after perturbing. re perturbation coefficients. While ***I*** qual to 30%, the *∆x*,*∆y*,*∆w*, *∆h* re in the range of [-0.3, 0.3]. Note that Eq.4 is performed on every bounding box in the training data.

**Figure 7:**
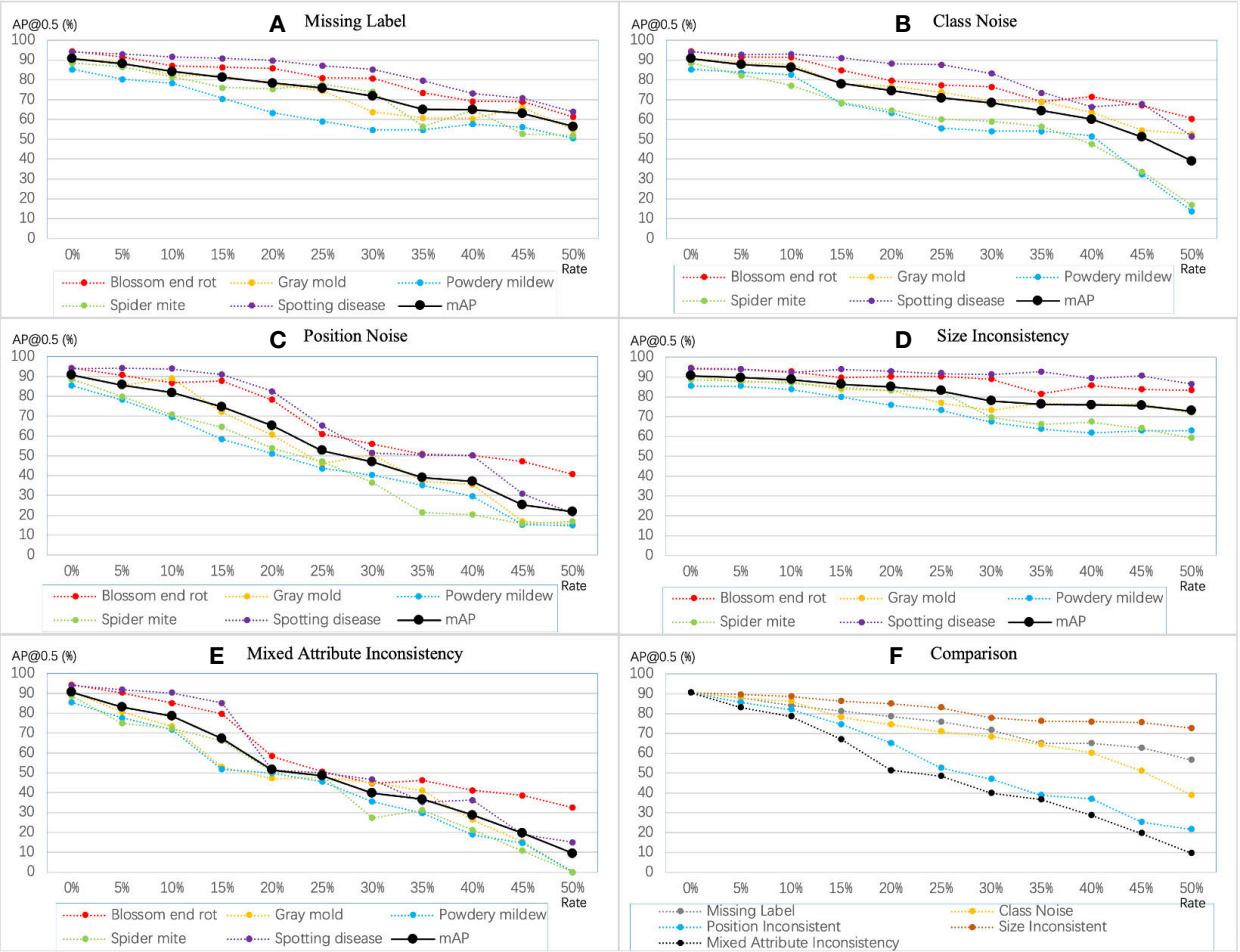
Sensitivity of YOLO-v5 extra-large model to different types of inconsistency. The mAP denotes and mAP@0.5.

As shown in **Figure 7**, the training accuracy linearly decreases with the increasing level of annotation inconsistency but varies among different types of inconsistency. Our observations are consistent with those observed in related work on noise. In the five types of inconsistency, positional inconsistency devastated the model’s performance. Conversely, the model is less sensitive to size inconsistency and missing labels, but the accumulated size inconsistency and missing label can still cause severe problems for the model. From the decreasing tendency, diseases with fewer instances are more susceptible to inconsistency. For example, when the mixed attribute inconsistency continued to increase, the average precision of gray mold, powdery mildew, and spider mite diseases dropped from around 90% to 0.

### 4.4 Inconsistency from data augmentation

**Figure 8:**
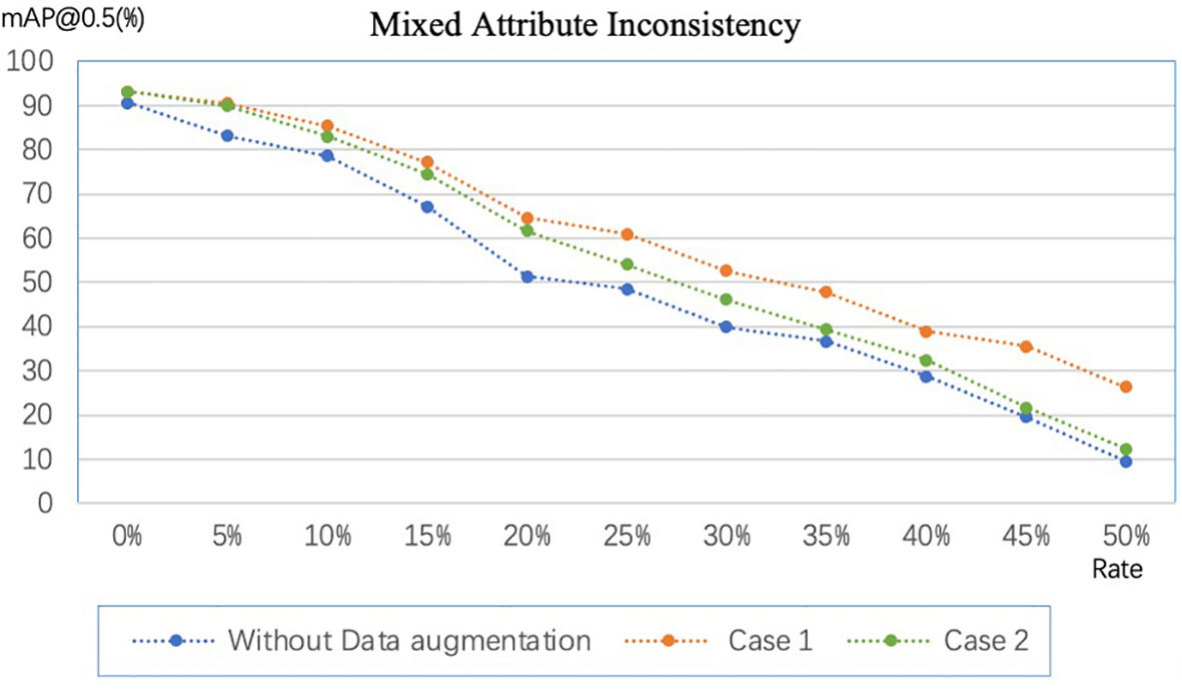
Comparison of data augmentation schemes in different scenarios. Case 1: expand the image and then annotate the dataset. Case 2: annotate the dataset first and then expand the dataset.

In section 3.4, we discussed label inconsistencies caused by data augmentation. **Figure 8** shows the experimental results, where Case 1 and Case 2 correspond to the two cases in the previous section. For fairness of comparison, we implement data augmentation with the same amount and policies for Case 1 and Case 2. Data augmentation policies include horizontal flip, vertical flip, center symmetry, and specific rotation ± (9, 180°). The experimental results show that the mAP@0.5 of Case 1 is always higher than that of Case 2, which indicates Case 1 is more robust to random errors than Case 2. However, Case 1 cannot be adopted when the dataset is enormous because the labeling cost will linearly increase. On the other hand, data augmentation by flipping and rotating by a specific angle can improve the model’s performance on our dataset even if the label set is inconsistent.

In addition, random rotation can lead the size of the rotated box to deviate from the correct, causing size inconsistency. **Table 5** shows the results of operating random rotation, right-angle rotation, and flipping. The results show that the inconsistency caused by rotation affects the performance gain brought by data augmentation to a certain extent. In the range of 0°-45°, the larger the rotation angle, the smaller the performance gain obtained by the model.

**Table 5:** Comparison of different rotation angles for data augmentation in Case 2.

Category	OriginalDataset	Specific rotation (±90°, 180°)	Random rotation (0°-5°)	Random rotation (0°-15°)	Random rotation (0°-30°)	Random rotation (0°-45°)
Blossom end rot (%)	94.3	**95.3**(+1.0%)	95.1 (+1.0%)	94.9 (+0.6%)	94.0(-0.3%)	94.2(-0.1%)
Gray mold (%)	90.8	**95.7**(+4.9%)	**95.7** (+4.9%)	94.1 (+3.3%)	92.4 (+1.6%)	91.4 (+0.6%)
Powdery mildew (%)	85.3	**90.2**(+4.9%)	89.5 (+4.2%)	88.7 (+3.4%)	87.5 (+2.2%)	87.0 (+1.7%)
Spider mite (%)	88.6	**89.9**(+1.3%)	**89.9** (+1.3%)	89.6 (+1.0%)	89.1 (+0.5%)	88.8 (+0.2%)
Spotting disease (%)	94.0	94.5(+0.5%)	**94.8** (+0.8%)	94.1 (+0.1%)	94.5 (+0.5%)	94.1 (+0.1%)
mAP@0.5 (%)	90.6	**93.1**(+2.5%)	93.0 (+2.4%)	92.3 (+1.7%)	91.5 (+0.9%)	91.1 (+0.5%)

The original dataset denotes the dataset without data augmentation. Bold fonts indicate the best performance in comparative experiments (Maximum).

### 4.5 Visualization

**Figure 9:**
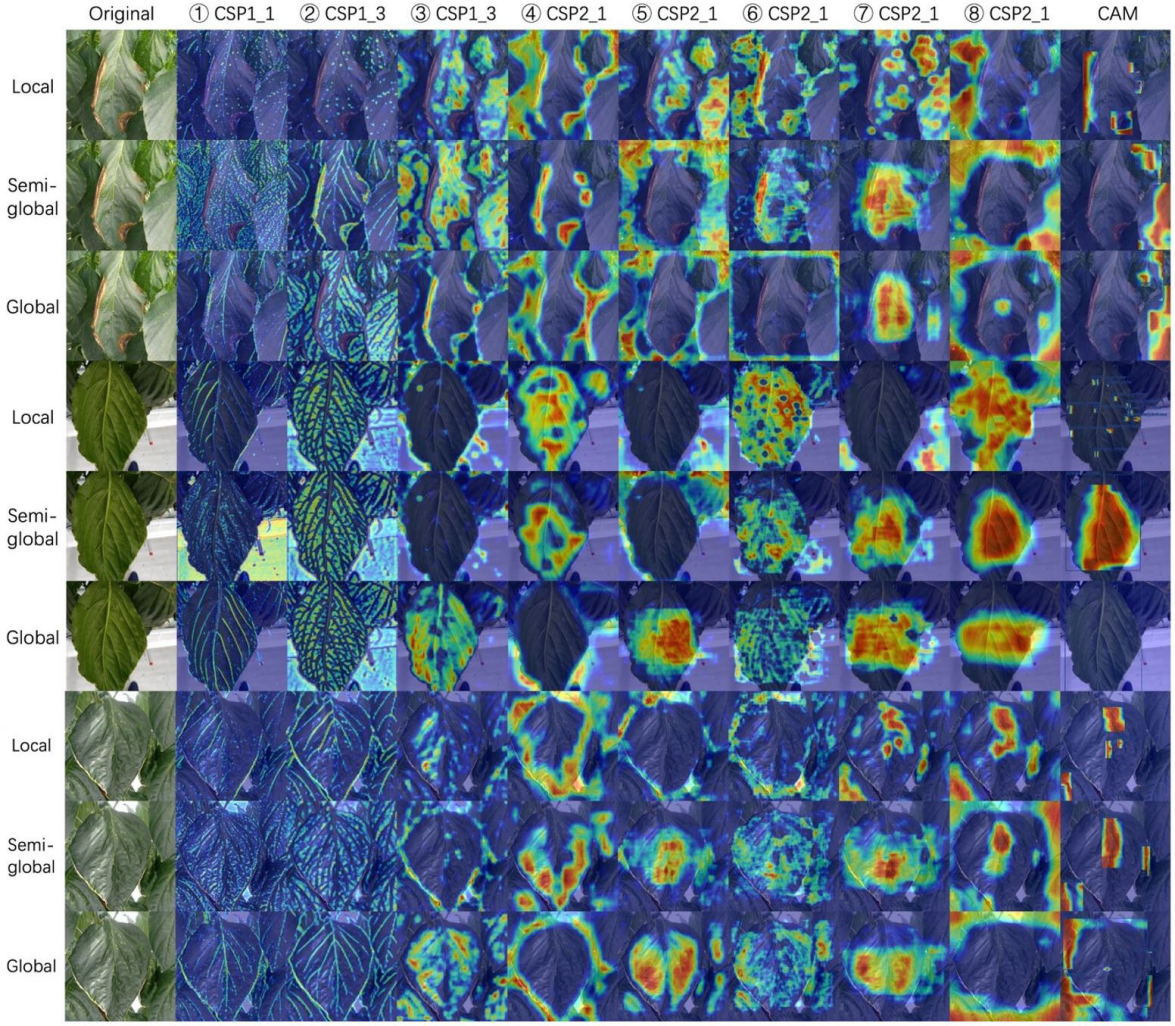
Class activation maps for three annotation strategies. We visualize all the CSP modules in the YOLO-5-x model from left to right. Rows 1-3, 4-6, and 7-9 show a sample of gray mold, powdery mildew, and spider mite, respectively. Class activation map (CAM) shows the output of the prediction layer. The red areas represent the areas that the model focuses on. From red to blue, the level of attention gradually decreases. Best viewed in color.

The community often criticizes the interpretability of CNNs, since these networks usually look like complicated black boxes. Therefore, the interpretability of CNNs has received extensive attention. For example, the class activation map (CAM) (Selvaraju et al., 2017; Muhammad and Yeasin, 2020; Wang et al., 2020; Jiang et al., 2021) focuses on making sense of what a model learns from the data or why it behaves poorly in a given task. Eigen-CAM (Muhammad and Yeasin, 2020) has proven to be a very efficient and convenient visualization method, which takes advantage of the principal components to improve the weights. It works with all CNN models without the need to modify layers or retrain models. More conveniently, Eigen-CAM can visualize the activation map of any layer in the neural network, which helps us understand the feature extraction of different layers. As shown in **Figure 9**, we compare the class activation maps learned by the YOLO-v5-x model under different annotation strategies. Cross-Stage-Partial-connections (CSP) is a crucial component of YOLO-v5 to implement a feature pyramid. As **Figure 6** shows, CSP modules No. 2, 3, 4, and 5 connect the backbone to the bottleneck, and CSP modules No. 6, 7, and 8 connect to the prediction layer. Therefore, we extracted the output of all CSP modules in the YOLO-v5 model for visualization to understand what the CNN learns.

**Figure 9** shows the visualization results of YOLO-v5-x model learning on datasets with different annotation strategies. We observed that the first two CSP modules can almost learn the edges and veins of leaves for all strategies. CSP modules 3, 4, and 5 seem more inclined to capture global features, leading to the features not being activated under the local labeling strategy. The last three CSP modules are connected to the output layers, which extract higher-level semantic features. In the local label strategy, the red areas are very scattered, which indicates that the model can focus on local lesions under this strategy. However, for boundary-inseparable diseases (refer to row 4 and row 7 of **Figure 9**), it is difficult for the model to accurately regress the location of multiple small lesions in the prediction layer because it cannot demarcate the boundaries of symptoms. It leads to low performance. On the contrary, for boundary-separable diseases (refer to row 1 of **Figure 9**), the local label strategy can accurately return the location of the disease. In addition, under the global label strategy, the features of the output layer are not activated. Although the model regresses a global bounding box to avoid the problem of boundary demarcation, the model does not pay more attention to the lesion area, which will lead to the model will miss detection if the lesions are small (refer to the last column of **Figure 9**). Therefore, the symptom-adaptive label strategy is used on our dataset, which enables the model to achieve good performance for each disease detection.

## 5 Discussion

**Figure 10:**
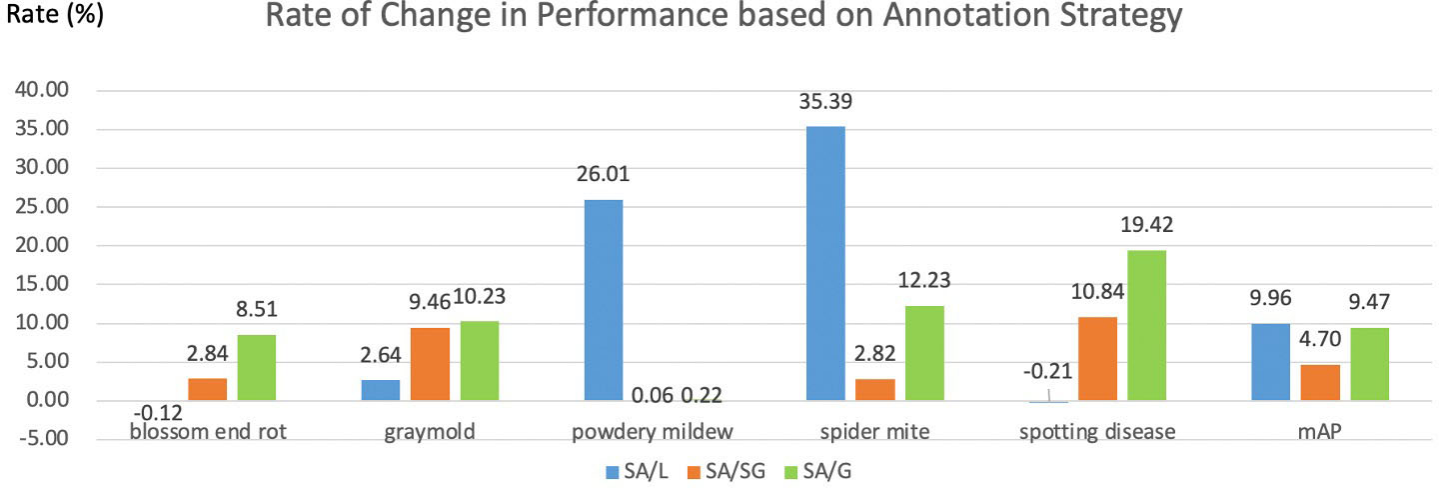
Rate of change in performance based on annotation strategy. L, SG, G, and SA denote local, semi-global, global, and symptom-adaptive respectively. The data comes from the YOLO-v5-Extra-large in **Table 3B**.

**Which annotation strategy is recommended?** In this part, we compare the pros and cons of four annotation strategies. The local label strategy has advantages that it can maximize the inter-class distance and reduce the intra-class distance. Meanwhile, more instances can be obtained from a local label strategy when the dataset is limited. Thus, the local label is often adopted in plant disease detection tasks. A local label can also support deeper tasks. For example, the severity of the disease can be judged by calculating the ratio of the disease area to the whole leaf. However, it is difficult for even humans to annotate precisely for boundary-inseparable diseases, which reduces the model’s performance. Therefore, a semi-global labeling scheme is a peaceful solution for these specific categories. We also visualize the rate of change in performance based on annotation strategy in **Figure 10**. As shown in **Figure 10**, symptom-adaptive strategy shows 10.76%, 5.10%, and 9.82% better than local strategy, semi-global strategy, and global strategy respectively. The symptom-adaptive annotation strategy achieves the best performance in almost all disease categories, which is a way to trade off annotation cost and the model’s performance. Although related works (Hughes and Salathé, 2015; Liu et al., 2017; Wiesner-Hanks et al., 2018; Parraga-Alava et al., 2019; Li et al., 2020; Singh et al., 2020; Fenu and Malloci, 2021) that employ a global labeling strategy have also achieved good results, we observe that each leaf of these datasets contains only one disease. Therefore, the global label strategy is a good choice for relatively simple datasets. As for a complex dataset containing multiple diseases on one leaf, we do not advocate the global labeling strategy. Through visualization analysis, we found that when there is only a single lesion on the leaf, it is tough for CNN to focus on smaller lesion areas under the global label strategy. Furthermore, annotating a leaf with more than one disease is unavailable under a global label strategy. We cannot give the best solution due to the different subsequent tasks and datasets. However, we recommend a symptom-adaptive label strategy for plant disease detection, mainly consisting of local and semi-global label strategies. In addition, it allows multiple diseases to be annotated on the same leaf while reducing annotation costs to a certain extent.

**Annotation consistency.** Annotation consistency is an essential indicator in evaluating the quality of data. Li Y et al. (Cubuk et al., 2019) concluded that limited good data could beat a lot of bad data. In our work, we found that datasets with inconsistent labels (bad data) caused a much more significant drop in performance than missing labels (limited good data), which is consistent with their conclusion. Although achieving 50% of any inconsistency levels is almost impossible, it is possible to reach 15%. **Figure 7** shows that the model’s performance decreased to less than 70%, with the mixed attribute inconsistency level increasing to 15%. In contrast, with 30% of the labels missing, the model’s performance is still above 70%. Therefore, we argue that an inconsistent label may significantly impact the network model more than a missing label. Besides, the results in (Liu et al., 2022) show that the CNN method can achieve the same performance as a clean dataset on a dataset with 10% label noise with advanced annotation correction techniques. Nonetheless, we still recommend that annotators strive to improve the consistency and accuracy during the annotation process rather than using correction techniques directly.

**Data augmentation by random rotation.** The result in **Table 5** indicated that when the size inconsistency caused by the rotation angle is small (the rotation angle is slight), data augmentation can achieve considerable performance gains, which is also the logic behind controlling the rotation angle in a detection task. While for classification and segmentation tasks, arbitrary rotations do not cause size inconsistencies since the labels are either image-level or pixel-level in these tasks. The target’s physical orientation also needs to be considered for larger rotation angles. In our dataset, however, since the leaves are oriented arbitrarily, the physical orientation does not need to be considered. As a result, we can use multiples of 90° of rotation, vertical flipping, and horizontal flipping, to avoid size inconsistency generated from a random rotation.

**Limitations and future work.** Due to the enormous annotation cost, there is currently a lack of research on annotation strategies in plant disease detection. Our work provides guidelines to some extent but still has limitations. For instance, our dataset only contains no more than 6,000 images. Hence, we cannot guarantee that we will still reach the same conclusions on large datasets with ten or one hundred times as many images as ours. In addition, the performance of deep learning models may be overestimated due to train-test data leakage (Nalepa et al., 2019). For a fair comparison, cross-validation is generally used to evaluate the model’s performance, which causes a huge computational cost, especially in deep learning methods. However, we focus on the impact of annotation strategy and consistency on the model’s performance, instead of evaluating the overestimation of the model performance. In addition, we emphasize the tendency of different types of consistency to lead to significant differences in model performance rather than obtaining a precise value. Nevertheless, to a certain extent, our work still reflects annotation strategy’s and consistency’s considerable impacts on deep learning models. In this work, we get a low-quality dataset by perturbing clean bounding boxes. Our future work will focus on automatically repairing the errors in the data labels and turning the low-quality data into high-quality data. Furthermore, we observed that disease symptom textures might be the same or similar across plants, while backgrounds (leaf vein, fruit, etc.) are different. Using different plant datasets to solve the problem of open-set domain adaptation across plants is also the focus of our future work.

## 6 Conclusion

The detection of plant diseases using digital images is a challenging task. Deep learning techniques seemingly can adequately address most of the technical challenges associated with plant disease classiffication. In this work, we argue that the model’s performance can be further improved by optimizing the annotation strategy instead of increasing the model’s complexity. Symptom-adaptive annotation strategy improves the feature representation ability of the model by improving the annotation consistency. Simultaneously, it can also reduce annotation costs and requirements for labelers. Moreover, analyzing annotation inconsistency in advance is necessary for plant disease detection. Experiments demonstrated that the impacts of inconsistency are severe in many circumstances. Compared with other kinds of inconsistency, position inconsistency is more damaging than class noise. The size inconsistency is usually less harmful but still could lead to a slight reduction in the performance of learning algorithms. Furthermore, data augmentation by rotating at random angles can cause size inconsistency, which affects the performance gain brought by data augmentation to a certain extent. Therefore, the random rotation should be used with caution to avoid size inconsistencies. We found that annotating the same instance multiple times can offset the impact of random errors when the inconsistency is unavoidable, but the cost will significantly increase. In addition to the above work, we also emphasized the interpretability of our methods. We explained the high efficiency of the symptom-adaptive strategy through visualization technics. With these conclusions, instead of adopting some ‘blind’ annotation strategies, noise handling mechanisms, and data augmentation policies, interested readers can design their own inconsistency handling approaches to enhance data quality from their perspectives.

## Data availability statement

The original contributions presented in the study are included in the article/supplementary materials. Further inquiries can be directed to the corresponding authors.

## Author contributions

JD designed the method, performed the experiments, and wrote the manuscript. DP and SY advised in the design of the system and analyzed the annotation strategies to find the best method for effcient plant disease detection. JL and AF provided support in the data collection and proofreading article. MX provided suggestions on perturbing the bounding box and the discussion on visualization. ML provides partial datasets and domain knowledge of plant diseases. All authors contributed to the article and approved the submitted version.
